# Extra-articular Fractures of the Scapula

**DOI:** 10.1055/s-0046-1818599

**Published:** 2026-05-07

**Authors:** Vincenzo Giordano, Robinson Esteves Pires, Pedro José Labronici

**Affiliations:** 1 Prof. Nova Monteiro Orthopedics and Traumatology Service , Hospital Municipal Miguel Couto, Rio de Janeiro, RJ, Brazil; 2 Clínica São Vicente, Rede D'or São Luiz, Rio de Janeiro, RJ, Brazil; 3 Locomotor System Department, School of Medicine, Universidade Federal de Minas Gerais (UFMG), Belo Horizonte, MG, Brazil; 4 Hospital Felício Rocho, Belo Horizonte, MG, Brazil; 5 Department of Orthopedics and Traumatology, School of Medicine, Universidade Federal Fluminense (UFF), Niterói, RJ, Brazil; 6 Prof. Dr. Donato D'Ângelo Orthopedics and Traumatology Service, Hospital Santa Teresa, Petrópolis, RJ, Brazil

**Keywords:** fracture fixation, scapula, shoulder fractures, escápula, fixação de fratura, fraturas do ombro

## Abstract

Extra-articular scapular fractures include those involving the coracoid process, the acromion, the scapular spine, and the inferior angle of the scapula. These injuries are uncommon, so they represent a therapeutic challenge. Historically, nonoperative treatment was the standard for these fracture types, although the outcomes were heterogeneous and often characterized by persistent pain, limited mobility, and scapulothoracic dyskinesis. Advances in imaging, surgical techniques, and implant quality have expanded surgical indications, demonstrating good medium- and long-term functional outcomes. In the current update article, the authors present the most recent literature on each of these scapular-fracture patterns and outline the current treatment recommendations.

## Resumo

As fraturas extra-articulares da escápula incluem aquelas que acometem o processo coracoide, o acrômio, a espinha e o ângulo inferior da escápula. São lesões pouco frequentes, de modo que representam um desafio terapêutico. Historicamente, o tratamento não cirúrgico era considerado padrão para esses tipos de fraturas, embora os resultados fossem heterogêneos, frequentemente marcados por dor persistente, limitação da mobilidade e discinesia escapulotorácica. Atualmente, com a evolução dos métodos de imagem, das técnicas operatórias e da qualidade dos implantes, as indicações cirúrgicas têm se ampliado, mostrando bons resultados funcionais no médio e longo prazos. Neste artigo de atualização, os autores apresentam a literatura mais recente referente a cada um desses padrões de fratura da escápula e expõem as recomendações de tratamento.

## Introduction

Scapular fractures are rare, representing less than 1% of all fractures and approximately 5% of shoulder-girdle fractures. 1 2 Regarding this low incidence, extra-articular fractures of the coracoid process, acromion, body, and spine of the scapula represent the majority of the cases, often with favorable functional outcomes in approximately 80% of the patients undergoing non-surgical treatment. 2 3 This outcome results from the shoulder's wide range of motion, which enables the maintenance of daily living activities even in the presence of some degree of posttraumatic deformity. 3 However, some patients complain of persistent pain and limited shoulder mobility, with a significant impact on the function of the affected upper limb. In such cases, it is necessary to define more specific parameters for the proper identification of which extra-articular scapular fractures have benefited from surgical treatment.

Recent advances in imaging techniques have improved morphological characterization and enhanced the accuracy of evaluating deviations. As a result, surgical treatment is now more frequently indicated for selected fracture patterns of the coracoid process, acromion, spine, and inferior angle of the scapula. 2 4 These indications are considered, in particular, to prevent dysfunction of the superior suspensory complex of the shoulder (SSCS), subacromial space invasion, rotator cuff impairment, and/or restrictions in scapulothoracic mobility. 2 4 5 6 7

In the present article, we review the surgical indications and present the main current approaches and fixation options for fractures of the coracoid process, acromion, spine, and inferior angle of the scapula.

## Acromion Fractures

Acromion fractures are rare, accounting for about 8% of all scapular fractures. 2 8 The classic injury mechanism is direct trauma to the lateral surface of the shoulder, frequently occurring in association with other injuries to the shoulder girdle and/or ipsilateral hemithorax. More rarely, acromion fractures can occur after acromioplasty, being more commonly observed after arthroscopic surgery than the open procedure, or as a postoperative complication of total reverse shoulder arthroplasty (TRSA), resulting from biomechanical changes and arm-length discrepancy. 2 8 9 Diagnosis can be a challenge, and it requires a high degree of suspicion on the part of the surgeon whenever the patient complains of intense shoulder pain after direct trauma to the lateral aspect of the shoulder. Radiographic studies should include the axillary view, considered the most sensitive to detect the fracture, and computed tomography (CT), including three-dimensional reconstruction (3D CT). In some cases, such as post-TRSA insufficiency fractures, magnetic resonance imaging may be necessary for better visualization of the fracture. It is worth noting that os acromiale, present in about 3% of the population, can be confused with a fracture line. 8

The treatment of acromion fractures remains controversial in the current literature, with no absolute surgical indications. The unique, complex, and slender anatomy of this structure, coupled with its multiple ligamentous and muscular attachments, makes its management a challenge. 2 Most authors recommend anatomical reduction and internal fixation for fractures with ≥ 1 cm of radiographic displacement, ipsilateral scapular fractures requiring surgery, or cases with multiple SSCS injuries to prevent painful pseudoarthrosis and protect the rotator cuff from subacromial impingement. 2 7

The adoption of a classification system aids in therapeutic decision-making. Among these classifications, the one by Kuhn et al. 10 stands out as the most useful, since it considers both fragment displacement and reduction of the subacromial space.

This classification system indicates non-surgical treatment for most stable, non-displaced fractures (Kuhn type I) and displaced fractures without reduction of the subacromial space (Kuhn type II) in patients with low functional demands. These cases require rigorous clinical and radiographic follow-up due to the risk of late and progressive displacement, especially in types IB and II. 10 The recommended treatment consists of using a simple sling for 6 to 8 weeks, followed by assisted passive mobilization from the third week on, and assisted active mobilization after the sixth week. 11

Although in theory the use of an abduction sling may reduce the lever arm of the deltoid muscle, and thus decrease the risk of secondary fracture displacement, there is no clinical evidence to support its superiority over a simple sling. 2 8 12 Sling removal should occur after the observation of radiographic or tomographic signs of fracture healing. 11 Surgical treatment is indicated for other fracture types, including type IB in high-demand patients. 10 11 The surgical approach should follow Langer's (cleavage) lines of tension, preferably just above the spine of the scapula, as a horizontal posterolateral incision. 13 If there is an associated fracture of the glenoid fossa or neck or of the scapular body, the Brodsky approach with proximal curved extension is a good alternative. 2 When a wider exposure with medial extension is necessary, the suprascapular nerve must be identified and protected.

**Fig. 1:**
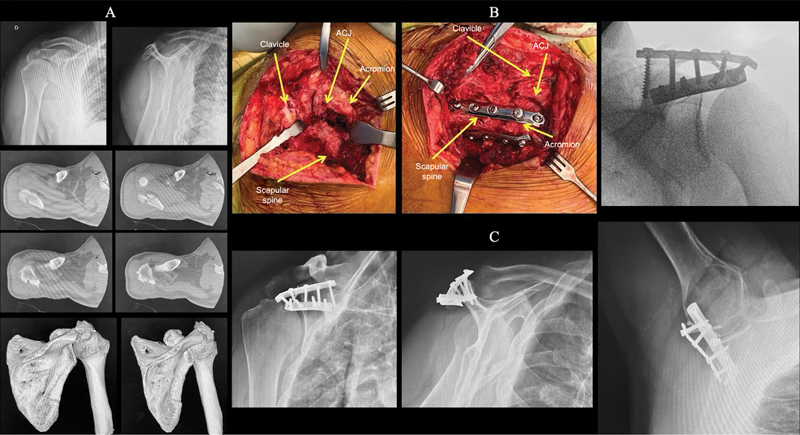
Right acromion fracture, Kuhn et al. 10 type III. ( **A** ) Radiographs and computed tomography scans showing the fracture and subacromial space invasion. ( **B** ) Intraoperative images of the fracture and double plate fixation. ( **C** ) One-year postoperative radiographs showing a consolidated fracture in good position and regular shoulder function. **Abbreviation:** ACJ, acromioclavicular joint.

Among the several fixation options described to treat acromion fractures, the preferred method uses a double orthogonal plate with locked, low-profile implants 11 ( Fig. 1 ). Several authors 2 7 8 13 14 15 have reported good outcomes with this method, highlighting the low rate of complications and the need for implant removal. In very distal fractures, in which the use of plates is not feasible, one must consider the tension band technique, as it enables adequate rotational control of the fragment. 7 8 13 In this fracture pattern, it is key to avoid using isolated screws, as they do not neutralize the deforming forces exerted by the deltoid muscle and the weight of the upper limb itself. 11 Before closing the surgical wound, it is crucial to perform a radiographic control, including the anteroposterior view with a 30° caudal inclination (Rockwood view), to ensure the lack of penetration of osteosynthesis screws into the subacromial space. 16

Passive and assisted active exercises should begin in the postoperative period according to tolerance, keeping abduction and flexion limited to 50°. The patient must use a simple sling for 1 to 2 weeks. Mobility increases progressively during the first 6 weeks, with radiographic checks in the first, third, and sixth weeks. Isometric exercises with low-resistance elastic bands should start after the sixth week. 11

## Coracoid Fractures

Coracoid fractures account for approximately 3 to 13% of all scapular fractures. 2 7 11 17 18 These injuries often result from high-energy traumas and are frequently associated with complex lesions in other parts of the scapula and in adjacent structures, especially the acromioclavicular joint, the lateral region of the clavicle, and the proximal humerus. 19

However, less frequently, and often undiagnosed at the initial assessment, fractures of the coracoid process can occur due to indirect trauma, especially after anterior shoulder dislocation. 19 Although stress fractures are even rarer, reports describe them in sports such as shooting, cricket, and golf. 19

The complex and variable three-dimensional orientation of the coracoid process hinders its radiographic visualization. As such, traditional shoulder trauma series views may not enable an accurate diagnosis. 18 19 20 Therefore, in addition to the trauma series, it is recommended to obtain specific orthogonal radiographs of the coracoid pillar, such as the incidence described by Bhatia, which improve the identification of fractures of the superior and inferior pillars, as well as the junctional region of the coracoid process. 11 20 To obtain these views, the patient remains standing or in the supine position, with the cassette resting on the posterior aspect of the affected shoulder.

Inferior coracoid pillar (ICP) visualization requires tilting the radiographic beam 30° to 40° cephalad, aligning it perpendicularly to the sagittal plane of the coracoid process. Additionally, an axial angulation from lateral to medial (20°–30°) is applied, centering the beam over the tip of the coracoid process.

Superior coracoid pillar (SCP) visualization requires tilting the affected shoulder 30° posteriorly, positioning the scapula parallel to the cassette, similarly to the Grashey view. Then, the beam is angled from 30 to 40° cephalad and 20 to 30° medial to lateral in the axial plane, also centered over the tip of the coracoid process. 11 20 In addition to radiographs, the evaluation must include supplementary CT scans with 3D reconstructions and bone subtraction of adjacent structures. 17

Therapeutic decision-making should use the Bartoníček et al. 17 classification system, as it considers the anatomical position of the fracture, and the Ogawa et al. 19 21 classification system, which relies on the relationship between the fracture line and the coracoclavicular ligament. 11

In isolated fractures of the coracoid process with minimal or no displacement, the non-surgical treatment may yield good outcomes, even when the conformation of the coracoacromial arch presents slight alterations. 21 In a systematic review of 97 studies 19 including 197 patients 71% of the isolated fractures classified as type I by Ogawa were managed non-surgically, with favorable outcomes even in cases of nonunion.

For these cases, we recommend a simple sling for 6 to 8 weeks, starting with assisted passive mobilization from the third week and assisted active mobilization after the sixth week. 11 Active flexion of the ipsilateral shoulder and elbow should be avoided in the initial period, as the traction exerted by the short head of the biceps may displace the fragment. Sling removal requires radiographic or tomographic confirmation of consolidation.

Indications for the surgical treatment include fractures located proximally to the coracoclavicular ligament, displacement ≥ 1 cm on imaging evaluation, or the presence of multiple SSCS lesions. 2 7 17 21 The procedure aims at preserving the configuration of the coracoacromial arch and maintaining continuity between the clavicle and scapula.

The approach to most coracoid fractures can use a direct vertical incision, following Langer's lines, initiated on the superior aspect of the clavicle, immediately above the coracoclavicular interval. 22 In cases associated with anterior or superior glenoid fractures, the Henry deltopectoral approach is the preferred method. 2 7 Less commonly, some non-displaced Ogawa type-I fractures may undergo percutaneous fixation, with or without arthroscopic assistance. 2

During fracture reduction, the operated upper limb should be placed in internal rotation and adduction to protect the brachial plexus. 2 Sutures applied to the conjoint tendon and threaded wires positioned orthogonally to the fragment aid in mobilization, facilitating reduction. 2 7 11 Following reduction, a Kirschner wire provides temporary fixation. 11

**Fig. 2:**
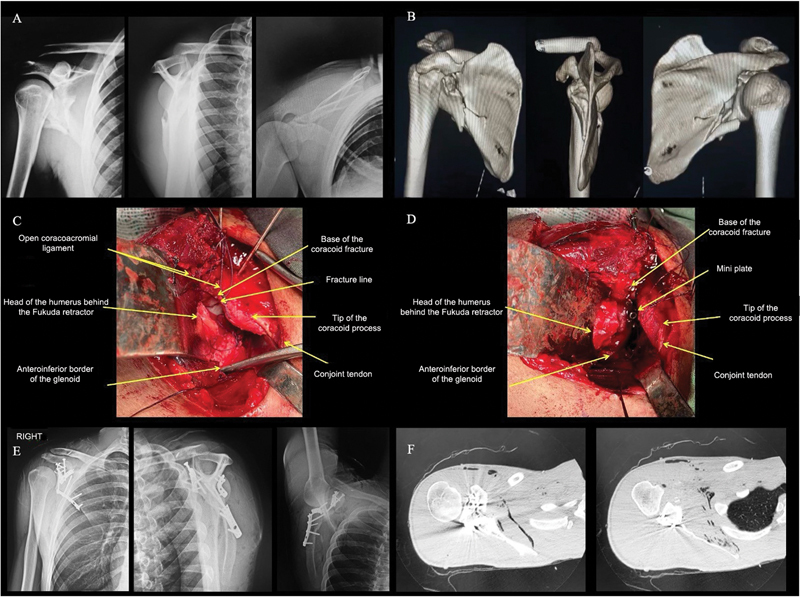
( **A** , **B** ) Trauma series radiographs and three-dimensional computed tomography (3D CT) scan of the right shoulder, showing a complex scapular fracture, with involvement of the coracoid process. The coracoid fracture was classified as type III (involving the coracoid process base) by Bartoníček et al. 17 and type II (distal to the attachment site for the coracoclavicular ligament) by Ogawa et al. 19 21 ( **C** , **D** ) Intraoperative images showing the fracture line after opening the coracoacromial ligament and fixating it with a 2.0-mm mini-fragment plate and a 3.5-mm traction screw. The coracoacromial ligament was repaired using an anchor screw following the coracoid fracture fixation. ( **E** , **F** ) Trauma series radiographs and axial CT scans of the right shoulder after surgery, showing fixation of all scapular fracture lines and quality of coracoid process and glenoid reduction.

Definitive fixation often uses a 3.5-mm cortical screw or, less frequently, a partially threaded cannulated screw of the same gauge. 2 11 Correct screw orientation is essential to ensure stability and prevent failure. Because the tip of the coracoid process is thin and curved in a hook shape, fixation initiated from this point is technically challenging; therefore, the ideal positioning is within the body of the coracoid process, towards its base and the neck of the scapula, a path known as the *coracoid tunnel* 2 11 17 23 ( Fig. 2 ).

In the postoperative period, the patient should begin passive and assisted active exercises, limiting abduction and flexion to a maximum of 50° and avoiding flexion against resistance of the elbow. A simple sling is used for 1 to 2 weeks for comfort. The range of motion gradually increases during the first 6 weeks, and clinical and radiographic monitoring occurs in the first, third, and sixth weeks. After the sixth week, isometric exercises with light resistance bands are initiated. 11

## Scapular Spine Fractures

Scapular spine fractures are relatively rare, accounting for less than 11% of all scapular fractures. Although the mechanism of injury is typically high-energy trauma, these fractures are frequent complications of TRSA. 5 24 25 26 The overall incidence of acromion and spine fractures associated with TRSA ranges from 0.8 to 10.2%. 5 24 25 26 In the current article, we will not address fractures secondary to TRSA because they have a different etiology and management compared with traumatic fractures. 25

In high-energy trauma, spinal fractures rarely occur in isolation, usually being associated with fractures of the vertebral body or glenoid. 11 23 In these cases, a high index of suspicion is essential, considering local pain, pain upon mobilization, and signs of dysfunction in the ipsilateral upper limb. 23

**Fig. 3:**
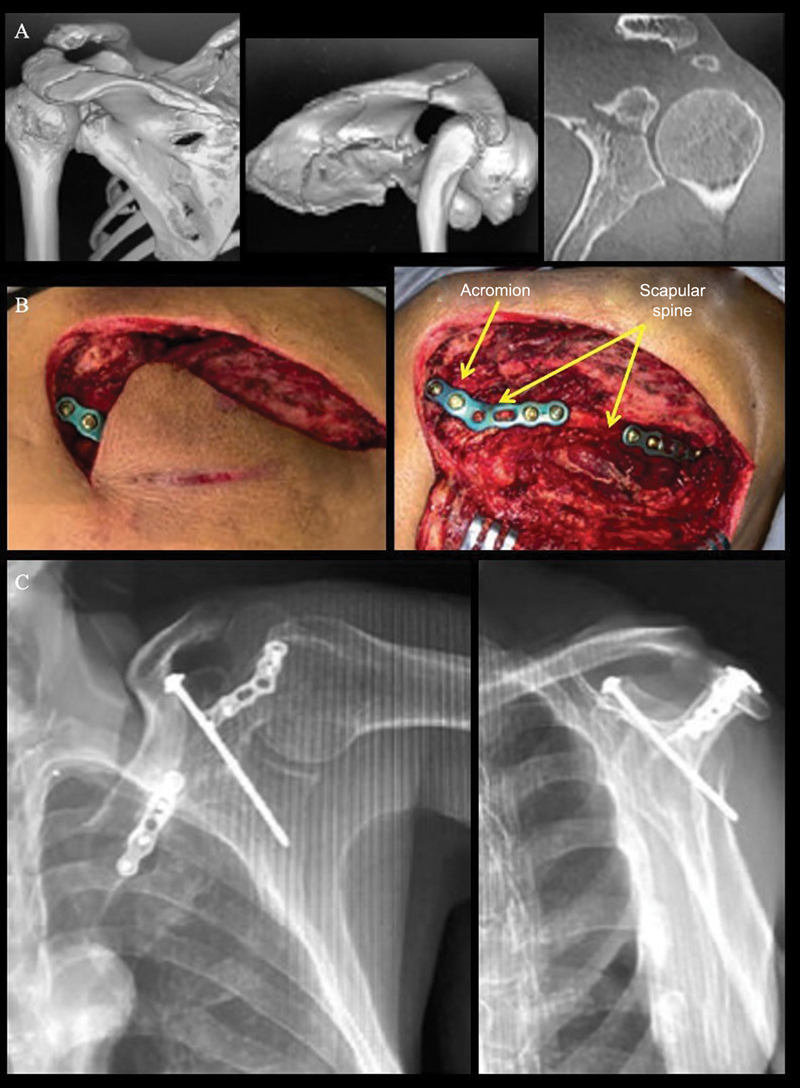
( **A** ) Three-dimensional and two-dimensional coronal computed tomography scans of the left shoulder, showing a segmental fracture of the spine of the scapula and the base of the coracoid process extending into the glenoid fossa. Note the horizontal S shape of the spine. ( **B** ) Intraoperative images showing a segmental fracture of the scapular spine and fixation with 2.4-mm dorsal locking plates. ( **C** ) Postoperative anteroposterior and lateral radiographs of the left scapula show fixation of the scapular spine and coracoid process fractures with a 2.5-mm traction screw. Note the quality of reduction of the coracoid process and glenoid fossa.

Treatment is predominantly surgical, and the patient's position and approach depend on associated scapular fractures. 11 23 The patient can be placed in the contralateral lateral decubitus or prone position, using mini approaches tailored to each type of fracture. Spinal fixation is preferably performed with smaller-diameter (2.0–2.7 mm) locking implants, although small fragment plates (3.5 mm) can be used when bone thickness is sufficient 27 28 ( Fig. 3 ).

In the postoperative period, the patient must use a simple sling for 6 weeks. Passive and assisted active exercises are initiated as tolerated, limiting abduction and flexion to a maximum of 50°. Range of motion exercises should increase progressively over the first 6 weeks. Clinical and radiographic follow-ups occur in the first, third, and sixth weeks. From the sixth week onwards, isometric exercises with low-resistance elastic bands are initiated. 11 23

## Inferior Angle of the Scapula Fractures

Isolated fracture of the inferior angle of the scapula is rare and usually occurs in lower-energy traumas due to avulsion of the periscapular muscles, such as the serratus anterior, teres major, rhomboid major, and latissimus dorsi. 29 30 In older patients with fractures resulting from marked osteoporosis, a high degree of diagnostic suspicion is necessary. In these cases, chest radiographs and shoulder trauma series often do not show the fracture, especially when there is no evident initial displacement. Thus, CT is essential in patients with intense localized pain, late bruising, or persistent dysfunction lasting longer than expected for an injury initially presumed as muscular. 30 31

Most cases, however, present an inferior angle fracture in the context of a multifragmentary fracture of the scapular body resulting from high-energy trauma. 4 30 Associated injuries to the ipsilateral hemithorax and shoulder girdle are common, including long thoracic nerve palsy, which can progress to winged scapula regardless of treatment. 4 31 32 In a systematic review of 17 articles, Mousafeiris et al. 32 observed anterior deviation in 64% of the cases and winged scapula in the same rate.

In isolated fractures of the inferior angle with little to no displacement and no anterior angulation, treatment is preferably non-surgical, with analgesia and the use of a simple sling for 6 to 8 weeks but performing pendulum exercises without limb elevation. 4 Clinical and radiographic evaluations should occur every 2 weeks to monitor for potential secondary displacement. A gradual increase in the shoulder range of motion begins in the sixth week. 4 32

**Fig. 4:**
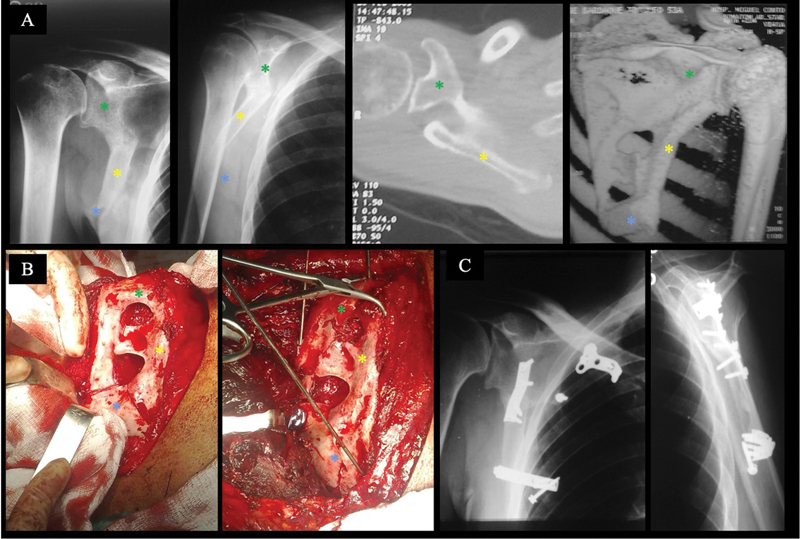
( **A** ) Anteroposterior and lateral radiographs of the scapula, two-dimensional axial CT scan, and 3D CT scan of the right shoulder, showing a complex, malunited fracture of the scapular body. Note the degree of deformity and shortening. Clinically, the patient presented with scapulothoracic dyskinesis. ( **B** ) Intraoperative images showing deformity of the scapular body and union defects. The fracture was approached using the classic Judet technique, with multiple osteotomies and derotation of the lateral border and inferior angle. ( **C** ) Anteroposterior and lateral radiographs of the left shoulder scapula taken 10 years after surgery. Note the complete correction of the body axis. Fixation was performed with small fragment plates. In the images, the three main fragments are highlighted with a green asterisk (*) for the superior fragment, including the neck and glenoid fossa, coracoid process, spine of the scapula, and acromion, an yellow asterisk (*) for the lateral fragment, including the lateral border of the scapula, and a blue asterisk (*) for the inferior angle of the scapula.

Surgical treatment is indicated for isolated fractures of the inferior angle with displacement or inclination of the medial border, as well as for fractures of the inferior angle associated with other fractures in the same bone. 23 The access route should follow Langer's lines, preferably in a horizontal or curvilinear orientation, at the level of the lower angle. 23 In the presence of an associated fracture of the scapular body, isolated or combined approaches are selected based on the fracture lines for fixation 30 ( Fig. 4 ).

In the initial postoperative period, a simple sling is used for 7 to 10 days until suture removal and proper pain control. Then, passive and active exercises of the ipsilateral upper limb are initiated. 4 23 31 32 33 In patients with unsuccessful non-surgical treatment or lack of initial diagnosis, malunion with scapular tilt can result in scapulothoracic dyskinesis. 34 35

## Final Considerations

Due to their low frequency, fractures of the coracoid process, acromion, spine, and inferior angle of the scapula continue to pose a challenge for surgeons treating scapulothoracic injuries. Advances in imaging quality and implant availability now enable the surgical treatment of selected displaced fractures that previously underwent conservative management, with the literature reporting good outcomes.

These surgical indications, however, are predominantly based on systematic reviews with low levels of evidence, retrospective case series studies, and isolated reports. Therefore, the information from these studies should be applied with caution when establishing individualized recommendations, always considering the patient's profile, their functional needs, the resources available at the institution, and the surgeon's experience in managing these complex fractures.
